# Association of Maintenance Hemodialysis with Transfer to Post-Acute Rehabilitation After Stroke: A Single-Center Retrospective Cohort Study

**DOI:** 10.3390/healthcare14193303

**Published:** 2026-10-03

**Authors:** Hidetaka Onda, Shoji Yokobori, Masato Miyauchi

**Affiliations:** 1 Department of Disaster and Emergency Medicine, Kochi University, Nankoku 783-8505, Japan; miyauchi@kochi-u.ac.jp; 2 Department of Advanced Emergency and Critical Care Center, Nippon Medical School Hospital, Tokyo 113-8603, Japan; shoji@nms.ac.jp

**Keywords:** maintenance hemodialysis, stroke, post-acute rehabilitation, discharge disposition, healthcare access, continuity of care

## Abstract

Background/Objectives: Maintenance hemodialysis (HD) is associated with poor outcomes after stroke, but its association with access to post-acute rehabilitation remains unclear. We examined rehabilitation transfer and outcomes according to maintenance HD status. Methods: This single-center retrospective cohort included consecutive stroke patients admitted to a tertiary emergency care center during 2015–2023. The primary outcome was rehabilitation transfer among in-hospital survivors. Rehabilitation eligibility and reasons for non-transfer were assessed in the HD group. Firth logistic regression evaluated rehabilitation transfer, and multivariable logistic regression evaluated in-hospital mortality and favorable functional outcome. Results: Among 1173 patients, 54 received maintenance HD. Rehabilitation transfer occurred in 2 of 33 HD survivors (6.1%) and 299 of 937 non-HD survivors (31.9%; risk ratio, 0.19; 95% confidence interval [CI], 0.05–0.62; *p* < 0.001). After adjustment using Firth logistic regression, maintenance HD remained associated with lower odds of rehabilitation transfer (adjusted odds ratio [aOR], 0.21; 95% CI, 0.04–0.65; *p* = 0.005). Of 12 rehabilitation-eligible HD patients, 2 (16.7%) were transferred; no facility providing both maintenance HD and inpatient rehabilitation could be secured for the other 10. Maintenance HD was associated with higher in-hospital mortality (aOR, 4.26; 95% CI, 2.20–8.24; *p* < 0.001) and lower odds of a favorable functional outcome (aOR, 0.38; 95% CI, 0.19–0.77; *p* = 0.007). Conclusions: Maintenance HD was associated with markedly lower rehabilitation transfer and poorer acute outcomes. Expanding access to facilities integrating dialysis and rehabilitation may improve continuity and equity of post-stroke care.

## 1. Introduction

Stroke is a leading cause of death and long-term disability worldwide. Access to appropriate post-acute rehabilitation is essential for functional recovery, social reintegration, and continuity of care after acute hospitalization. Early rehabilitation and continued inpatient rehabilitation in an appropriate post-acute setting are recommended according to neurological severity and functional status [1,2].

In Japan, 344,640 patients were receiving maintenance dialysis at the end of 2019, with a mean age of 69.1 years [3]. Patients receiving maintenance hemodialysis (HD) have a substantially higher risk of stroke and poorer clinical outcomes than the general population [4,5,6,7,8,9,10,11,12,13,14,15]. However, previous research has focused mainly on acute mortality and functional outcomes, and evidence regarding access to post-acute rehabilitation among stroke survivors receiving HD remains limited.

Access to post-acute rehabilitation depends not only on neurological recovery but also on health-system capacity, including the availability and capabilities of receiving facilities. Discharge destination is influenced by neurological severity, age, pre-stroke function, treatment, and social circumstances. Internationally, pathways from acute stroke care to post-acute rehabilitation vary according to the organization, availability, and eligibility criteria of inpatient rehabilitation and skilled nursing services [16,17,18]. For patients receiving maintenance HD, such health-system differences may be particularly relevant because access depends not only on rehabilitation needs but also on the availability of facilities capable of providing ongoing dialysis. Previous studies evaluated only patients who had already gained admission to rehabilitation facilities capable of continuing HD and reported either comparable functional improvement or poorer motor recovery than in patients not receiving HD [19,20]. These studies therefore could not evaluate patients who were eligible for rehabilitation but were unable to access an appropriate facility. Whether maintenance HD is associated with reduced transfer to post-acute rehabilitation after stroke, including through limitations in receiving-facility capacity, remains unclear.

To address this gap, we compared post-acute disposition and acute clinical outcomes between consecutive patients with stroke receiving and not receiving maintenance HD at a tertiary emergency care center. The primary objective was to compare transfer to post-acute rehabilitation among in-hospital survivors. We also compared overall disposition—transfer to rehabilitation, transfer for continued medical care, discharge home, or in-hospital death—and, within the HD group, assessed whether patients retrospectively judged eligible for post-acute rehabilitation were actually transferred and documented the reasons for non-transfer.

## 2. Materials and Methods

### 2.1. Study Design and Setting

This single-center retrospective cohort study was conducted at the Advanced Critical Care and Emergency Center of Nippon Medical School Hospital, a tertiary emergency facility that accepts critically ill patients, including those with severe stroke. Consecutive patients admitted between 1 January 2015 and 31 December 2023 were identified from an institutional clinical database. Information not available in the database was obtained through a review of the medical records. This study was reported in accordance with the Strengthening the Reporting of Observational Studies in Epidemiology (STROBE) statement.

### 2.2. Study Population and Data Collection

Patients transported to the center and diagnosed on admission with cerebral infarction, intracerebral hemorrhage, or subarachnoid hemorrhage were eligible if their hemodialysis status could be ascertained. Patients with other admission diagnoses were excluded. Patients receiving maintenance hemodialysis before stroke onset constituted the HD group, and all remaining patients constituted the non-HD group. For patients receiving maintenance HD, dialysis duration before stroke onset was ascertained from the medical records.

The collected variables included age, sex, Glasgow Coma Scale (GCS) score and systolic and diastolic blood pressure on arrival, stroke subtype, surgical intervention, length of hospital stay, Glasgow Outcome Scale (GOS) at discharge, and final discharge or transfer destination. For patients receiving maintenance HD, pre-stroke functional status was determined from medical records describing baseline activities of daily living before stroke onset and recorded using the modified Rankin Scale (mRS). Stroke subtype was classified according to the admission diagnosis as cerebral infarction, intracerebral hemorrhage, or subarachnoid hemorrhage. Surgical intervention was treated as a binary variable.

### 2.3. Outcomes and Definitions

The primary outcome was transfer to post-acute rehabilitation among in-hospital survivors. Final disposition among all patients was classified into four mutually exclusive categories: transfer to rehabilitation, transfer for continued medical care, discharge home, or in-hospital mortality. Among in-hospital survivors, post-acute disposition was classified as transfer to rehabilitation, transfer for continued medical care, or discharge home.

Transfer to rehabilitation was defined as transfer to another hospital primarily to continue inpatient rehabilitation after completion of acute stroke care. Transfer for continued medical care was defined as transfer to another department within the same hospital or to another hospital primarily for continued medical treatment or long-term care rather than post-acute rehabilitation.

Secondary outcomes were post-acute disposition among in-hospital survivors, in-hospital death, favorable GOS outcome, length of hospital stay, and overall final disposition. GOS at discharge was categorized as good recovery, moderate disability, severe disability, vegetative state, or death. Good recovery or moderate disability was defined as a favorable functional outcome.

Because rehabilitation eligibility was retrospectively assessed only in the HD group, two additional exploratory analyses used discharge GOS as a proxy for functional status potentially compatible with post-acute rehabilitation. These analyses compared rehabilitation transfer between the HD and non-HD groups among (1) patients with good recovery or moderate disability after excluding those discharged home and (2) patients with moderate disability after excluding those discharged home.

### 2.4. Retrospective Assessment of Rehabilitation Eligibility in the HD Group

For all patients receiving maintenance HD, the medical records were reviewed retrospectively, and post-acute status was classified as eligible for rehabilitation, not eligible for rehabilitation, or able to be discharged home. Eligibility for post-acute rehabilitation required both a need for continued inpatient rehabilitation and medical and neurological suitability for transfer after completion of acute treatment.

A single investigator board-certified in emergency medicine and neurosurgery assessed eligibility using the documented neurological status, general medical condition, activities of daily living, and discharge-planning records. Actual disposition was determined during routine clinical care by a treating team of 3–5 physicians drawn from an attending physician group of approximately 10 physicians. For patients considered eligible for rehabilitation who were not transferred to rehabilitation, the same investigator reviewed the medical records and discharge-planning documentation to determine the documented reasons for non-transfer and whether a receiving facility capable of providing both maintenance HD and inpatient rehabilitation could be secured. Among patients receiving HD who were retrospectively considered eligible for post-acute rehabilitation, the proportion actually transferred to rehabilitation was calculated.

### 2.5. Statistical Analysis

Continuous variables are presented as medians with interquartile ranges, and categorical variables as numbers and percentages. Continuous variables were compared using the Mann–Whitney U test. Categorical variables were compared using Pearson’s chi-squared test or exact tests, as appropriate. The four-category final-disposition distribution among all patients and the three-category post-acute disposition distribution among in-hospital survivors were compared between the HD and non-HD groups using exact tests for contingency tables. For rehabilitation transfer among all patients and among in-hospital survivors, transfer for continued medical care and discharge home among in-hospital survivors, as well as for the two proxy-defined rehabilitation-transfer analyses, unadjusted risk ratios and absolute risk differences with 95% confidence intervals were calculated. Risk ratios compared the HD group with the non-HD group. Confidence intervals for risk ratios were estimated using the score method, and those for risk differences were estimated using the Newcombe method. Risk differences were expressed in percentage points. To explore potential temporal changes in rehabilitation transfer during the study period, the cohort was divided into three 3-year periods (2015–2017, 2018–2020, and 2021–2023), and rehabilitation transfer rates among in-hospital survivors were descriptively compared according to maintenance HD status.

To evaluate the association between maintenance HD and rehabilitation transfer after adjustment for potential confounders, Firth’s penalized likelihood logistic regression was performed among in-hospital survivors. This approach was used to reduce small-sample bias and potential separation associated with sparse outcome data. The model included HD status, age, sex, GCS score on arrival, and stroke subtype. These covariates were selected based on their clinical relevance as potential confounders. The non-HD group and cerebral infarction served as the reference categories. Adjusted odds ratios with 95% confidence intervals were reported.

Multivariable logistic regression was performed to evaluate the associations of maintenance HD with in-hospital death and favorable GOS outcome. The models included HD status, age, sex, GCS score on arrival, and stroke subtype. These covariates were selected based on their clinical relevance as potential confounders. The non-HD group and cerebral infarction served as the reference categories. Adjusted odds ratios with 95% confidence intervals were reported.

Sensitivity analyses of in-hospital death and favorable GOS outcome were performed after excluding patients with subarachnoid hemorrhage and, separately, among patients with intracerebral hemorrhage alone. The analysis excluding subarachnoid hemorrhage included HD status, age, sex, GCS score on arrival, and stroke subtype. The analysis restricted to intracerebral hemorrhage included HD status, age, sex, and GCS score on arrival. As an additional sensitivity analysis, propensity score matching was performed to address baseline imbalances between patients receiving and not receiving maintenance HD. Propensity scores were estimated using age, sex, GCS score on arrival, stroke subtype, and systolic blood pressure. Patients were matched using 1:3 nearest-neighbor matching without replacement, with a caliper width of 0.2 standard deviations of the logit of the propensity score and exact matching on stroke subtype. Covariate balance was assessed using standardized mean differences (SMDs), with an absolute SMD < 0.10 considered indicative of adequate balance. Propensity score matching was performed separately in the overall cohort and among in-hospital survivors. Matched outcomes were analyzed using conditional logistic regression.

Analyses were conducted using complete records for the variables required for each analysis, and blood pressure comparisons used available cases. No imputation was performed. No a priori sample-size calculation was performed, and all eligible patients admitted during the study period were included. All tests were two-sided, and *p* < 0.05 was considered statistically significant. No adjustment for multiple comparisons was applied to the exploratory or sensitivity analyses. Statistical analyses were performed using R version 4.5.2 (R Foundation for Statistical Computing, Vienna, Austria).

### 2.6. Use of Artificial Intelligence

Artificial intelligence tools were used solely for English grammar and language editing. No artificial intelligence tools were used for study design, data collection, data analysis, data interpretation, the literature selection, figure preparation, or generation of scientific content. All authors reviewed and approved the final manuscript and accept full responsibility for its content.

## 3. Results

### 3.1. Study Population and Baseline Characteristics

**Figure 1:**
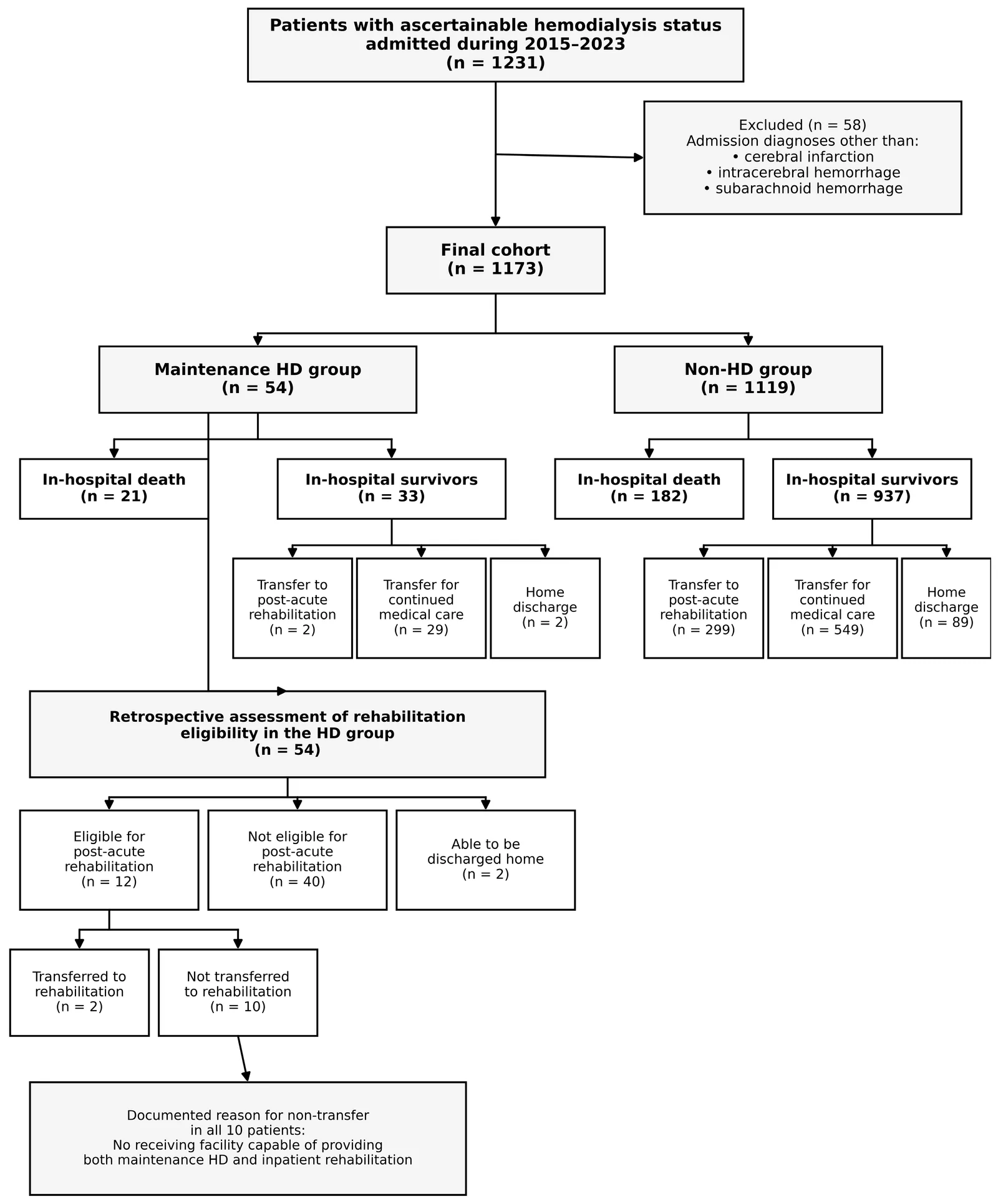
Study flow diagram and post-acute disposition according to maintenance hemodialysis status. Patients with ascertainable hemodialysis status admitted during 2015–2023 were screened. After exclusion of 58 patients with admission diagnoses other than cerebral infarction, intracerebral hemorrhage, or subarachnoid hemorrhage, 1173 patients were included in the final cohort. Clinical outcomes and post-acute disposition are shown separately for patients receiving maintenance HD and those not receiving HD. In the HD group, rehabilitation eligibility was additionally assessed retrospectively, and documented reasons for non-transfer among eligible patients were reviewed. Abbreviation: HD, hemodialysis.

Among 1231 patients with ascertainable hemodialysis status, 58 with admission diagnoses other than cerebral infarction, intracerebral hemorrhage, or subarachnoid hemorrhage were excluded. The final cohort comprised 1173 patients (Figure 1), including 54 receiving maintenance HD and 1119 not receiving HD. All patients in the HD group had received maintenance hemodialysis for more than 3 months before stroke onset, with the shortest dialysis duration being 2 years and 3 months. Overall, 269 patients had cerebral infarction, 574 had intracerebral hemorrhage, and 330 had subarachnoid hemorrhage. All patients receiving maintenance HD had been functionally independent before stroke onset (modified Rankin Scale score = 0).

Patients receiving HD were more frequently male than those not receiving HD (43/54 [79.6%] vs. 591/1119 [52.8%]; *p* < 0.001). Median age was 65.5 [interquartile range (IQR), 60.0–75.8] years in the HD group and 70.0 [56.0–79.5] years in the non-HD group (*p* = 0.252). Median systolic blood pressure on arrival was higher in the HD group (190 [168–220] vs. 163 [141–194] mmHg; *p* < 0.001), whereas diastolic blood pressure did not differ significantly between the groups (100 [87–113] vs. 92 [78–111] mmHg; *p* = 0.090). Systolic blood pressure was available for 53 of 54 patients in the HD group and 1114 of 1119 patients in the non-HD group; diastolic blood pressure was available for 53 of 54 and 1105 of 1119 patients, respectively. Median Glasgow Coma Scale (GCS) score on arrival was 6.5 [4–12] in the HD group and 8 [4–13] in the non-HD group (*p* = 0.313). No outcome or covariate data included in the multivariable models were missing.

In the HD group, 15 patients (27.8%) had cerebral infarction, 36 (66.7%) had intracerebral hemorrhage, and 3 (5.6%) had subarachnoid hemorrhage, compared with 254 (22.7%), 538 (48.1%), and 327 (29.2%), respectively, in the non-HD group. The overall distribution of stroke subtype differed significantly between the groups (*p* < 0.001). Surgical intervention was performed less frequently in the HD group than in the non-HD group (20/54 [37.0%] vs. 634/1119 [56.7%]; *p* = 0.005) (Table 1).

### 3.2. Access to Post-Acute Rehabilitation

Of the 54 patients receiving maintenance HD, 12 were retrospectively considered eligible for post-acute rehabilitation, 40 were considered ineligible, and 2 were considered able to be discharged home. Among the 12 patients considered eligible, only 2 (16.7%) were transferred to rehabilitation, whereas the remaining 10 (83.3%) were transferred for continued medical care. For all 10 eligible patients who were not transferred to rehabilitation, the medical records and discharge-planning documentation indicated that no receiving facility capable of providing both maintenance HD and inpatient rehabilitation could be secured (Supplementary Table S1).

**Figure 2:**
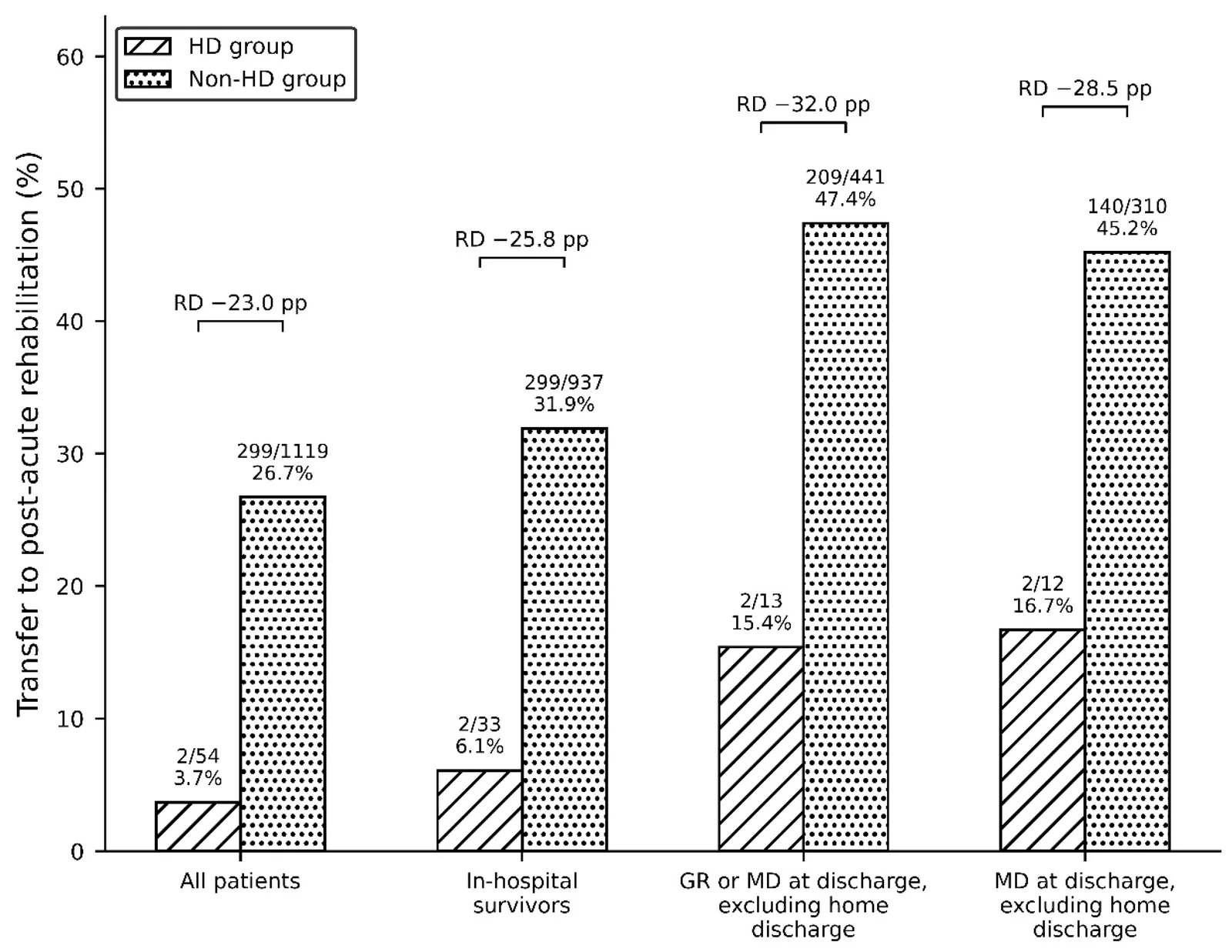
Transfer to post-acute rehabilitation according to maintenance hemodialysis status across the study populations. Transfer to post-acute rehabilitation was compared between patients receiving maintenance hemodialysis (HD) and those not receiving HD among all patients, in-hospital survivors, patients with good recovery or moderate disability after excluding those discharged home, and patients with moderate disability after excluding those discharged home. Values above the bars indicate the number transferred to rehabilitation divided by the total number in each analysis population, followed by the corresponding percentage. Risk differences (RDs), expressed in percentage points, are shown above each comparison. The corresponding 95% confidence intervals were −26.8 to −13.8, −31.2 to −12.0, −44.0 to −4.8, and −41.7 to 0.2, respectively. Abbreviations: GR, good recovery; HD, hemodialysis; MD, moderate disability; RD, risk difference.

Among the 970 in-hospital survivors, transfer to rehabilitation occurred in 2 of 33 patients receiving HD (6.1%) and 299 of 937 patients not receiving HD (31.9%). Transfer for continued medical care occurred in 29 of 33 HD survivors (87.9%) and 549 of 937 non-HD survivors (58.6%), whereas discharge home occurred in 2 of 33 (6.1%) and 89 of 937 (9.5%), respectively. The overall distribution of post-acute disposition differed significantly between the groups (*p* < 0.001) (Table 2; Figure 2).

Compared with survivors not receiving HD, those receiving HD were significantly less likely to be transferred to rehabilitation (risk ratio [RR], 0.19; 95% confidence interval [CI], 0.05–0.62; risk difference [RD], −25.8 percentage points; 95% CI, −31.2 to −12.0; *p* < 0.001) and significantly more likely to be transferred for continued medical care (RR, 1.50; 95% CI, 1.23–1.66; RD, 29.3 percentage points; 95% CI, 13.8–37.3; *p* < 0.001). The proportion discharged home did not differ significantly between the groups (RR, 0.64; 95% CI, 0.17–2.10; RD, −3.4 percentage points; 95% CI, −8.3 to 10.2; *p* = 0.762) (Supplementary Table S2). After adjustment for age, sex, GCS score on arrival, and stroke subtype using Firth’s penalized likelihood logistic regression, maintenance HD remained associated with lower odds of transfer to post-acute rehabilitation (adjusted odds ratio [aOR], 0.21; 95% CI, 0.04–0.65; *p* = 0.005).

The direction of the association was consistent across the additional analysis populations shown in Figure 2. Among patients with good recovery or moderate disability after excluding those discharged home, transfer to rehabilitation occurred in 2 of 13 patients receiving HD (15.4%) and 209 of 441 patients not receiving HD (47.4%) (RR, 0.32; 95% CI, 0.09–0.90; RD, −32.0 percentage points; 95% CI, −44.0 to −4.8; *p* = 0.025). In the more restrictive analysis limited to patients with moderate disability after excluding those discharged home, transfer to rehabilitation occurred in 2 of 12 patients receiving HD (16.7%) and 140 of 310 patients not receiving HD (45.2%) (RR, 0.37; 95% CI, 0.10–1.00; RD, −28.5 percentage points; 95% CI, −41.7 to 0.2; *p* = 0.073) (Figure 2; Supplementary Table S2). Rehabilitation transfer rates were also examined across three calendar periods. Among in-hospital survivors receiving HD, rehabilitation transfer occurred in 1 of 12 patients (8.3%) in 2015–2017, 1 of 10 (10.0%) in 2018–2020, and 0 of 11 (0%) in 2021–2023. The corresponding rates among patients not receiving HD were 135 of 401 (33.7%), 120 of 410 (29.3%), and 44 of 126 (34.9%), respectively, showing no clear temporal shift in rehabilitation transfer during the study period (Supplementary Table S3).

### 3.3. Overall Disposition and Clinical Outcomes

Among all patients, transfer to rehabilitation occurred in 2 of 54 patients receiving HD (3.7%) and 299 of 1119 patients not receiving HD (26.7%). Transfer for continued medical care occurred in 29 (53.7%) and 549 (49.1%), discharge home in 2 (3.7%) and 89 (8.0%), and in-hospital death in 21 (38.9%) and 182 (16.3%), respectively. The overall four-category final-disposition distribution differed significantly between the groups (*p* < 0.001) (Table 2).

Median length of hospital stay was 11.7 [IQR, 8.0–21.0] days in the HD group and 18.0 [7.0–29.0] days in the non-HD group (*p* = 0.101). Favorable Glasgow Outcome Scale (GOS) outcomes occurred in 15 of 54 patients receiving HD (27.8%) and 526 of 1119 patients not receiving HD (47.0%) (*p* = 0.007). In-hospital mortality occurred in 21 of 54 (38.9%) and 182 of 1119 (16.3%), respectively (*p* < 0.001).

In the HD group, GOS at discharge was classified as good recovery in 3 patients (5.6%), moderate disability in 12 (22.2%), severe disability in 16 (29.6%), vegetative state in 2 (3.7%), and death in 21 (38.9%). The corresponding numbers in the non-HD group were 205 (18.3%), 321 (28.7%), 283 (25.3%), 128 (11.4%), and 182 (16.3%), respectively. The overall GOS distribution differed significantly between the groups (*p* < 0.001) (Table 2).

After adjustment for age, sex, GCS score on arrival, and stroke subtype, maintenance HD was independently associated with higher in-hospital mortality (adjusted odds ratio [aOR], 4.26; 95% CI, 2.20–8.24; *p* < 0.001) and lower odds of a favorable functional outcome (aOR, 0.38; 95% CI, 0.19–0.77; *p* = 0.007) (Table 3).

### 3.4. Sensitivity Analyses

After exclusion of patients with subarachnoid hemorrhage, maintenance HD remained associated with higher in-hospital mortality (aOR, 3.98; 95% CI, 1.99–7.97; *p* < 0.001) and lower odds of a favorable GOS outcome (aOR, 0.40; 95% CI, 0.19–0.84; *p* = 0.016). In the analysis restricted to patients with intracerebral hemorrhage, maintenance HD was similarly associated with higher in-hospital mortality (aOR, 4.32; 95% CI, 1.98–9.44; *p* < 0.001) and lower odds of a favorable GOS outcome (aOR, 0.27; 95% CI, 0.09–0.82; *p* = 0.021) (Supplementary Table S4). Propensity score matching achieved adequate balance in the included covariates, with all absolute SMDs < 0.10 after matching (Supplementary Table S5). In the overall matched cohort, 53 patients receiving HD were matched to 159 patients not receiving HD. In-hospital mortality remained higher in the HD group (21/53 [39.6%] vs. 31/159 [19.5%]; matched OR, 2.71; 95% CI, 1.36–5.42; *p* = 0.005), whereas the association with favorable GOS outcome was attenuated and was no longer statistically significant (14/53 [26.4%] vs. 60/159 [37.7%]; matched OR, 0.53; 95% CI, 0.24–1.13; *p* = 0.101). Among propensity score–matched in-hospital survivors, rehabilitation transfer remained less frequent in the HD group than in the non-HD group (2/32 [6.3%] vs. 25/96 [26.0%]; matched OR, 0.20; 95% CI, 0.04–0.87; *p* = 0.032) (Supplementary Table S6).

## 4. Discussion

In this single-center cohort of patients with stroke admitted to a tertiary emergency care center, maintenance hemodialysis (HD) was associated with poorer acute outcomes and a markedly different post-acute care pathway. Among in-hospital survivors, 6.1% of patients receiving HD were transferred to rehabilitation, compared with 31.9% of patients not receiving HD, whereas 87.9% of HD survivors were transferred for continued medical care. Maintenance HD remained associated with lower odds of rehabilitation transfer after adjustment for age, sex, GCS score on arrival, and stroke subtype using Firth’s penalized likelihood logistic regression. More importantly, only 2 of 12 patients retrospectively considered eligible for post-acute rehabilitation were actually transferred to rehabilitation. For all 10 eligible patients who were not transferred to rehabilitation, no receiving facility capable of providing both maintenance HD and inpatient rehabilitation could be secured. The direction of the association was consistent in the exploratory analyses using discharge functional status as a proxy for rehabilitation eligibility, although the most restrictive analysis was imprecise. These findings extend previous reports of poor stroke outcomes in patients receiving HD [9,12,13,14] by suggesting that the need to continue HD may affect not only acute prognosis but also access to appropriate post-acute care.

Previous studies evaluating rehabilitation after stroke in patients receiving HD included only patients who had already been admitted to convalescent rehabilitation facilities capable of providing dialysis. One study reported functional improvement comparable with that of patients not receiving HD [19], whereas another demonstrated poorer recovery of motor and walking function [20]. These studies provide important information about rehabilitation effectiveness after successful admission but cannot evaluate patients who are unable to access an appropriate rehabilitation facility. In a previous historical cohort study conducted at a tertiary emergency and critical care center, implementation of an early rehabilitation program was associated with better functional outcomes and more frequent transfer to post-acute rehabilitation [21]. Inpatient rehabilitation can improve physical function in patients with end-stage kidney disease [22], can be safely delivered while maintenance HD is continued [23], and may be facilitated through coordination of dialysis and rehabilitation schedules [24]. Japanese guidelines recommend exercise therapy for patients receiving dialysis [25], and meta-analyses have demonstrated improvements in exercise capacity, muscle strength, physical function, and quality of life [26,27,28]. Collectively, this evidence indicates that maintenance HD itself should not be regarded as evidence of limited rehabilitation potential.

Despite this evidence, 10 of the 12 patients considered eligible for post-acute rehabilitation in the present study were transferred for continued medical care rather than rehabilitation. Frailty, sarcopenia, malnutrition [29], low physical activity [30], and other dialysis-related comorbidities may increase the complexity of rehabilitation and influence facility acceptance. Nevertheless, the documented proximate barrier in all 10 cases was the inability to secure a receiving facility capable of providing both maintenance HD and inpatient rehabilitation. This finding suggests that the transition from acute stroke care to post-acute rehabilitation may represent an important gap in the care pathway for patients receiving maintenance HD. It also distinguishes access to rehabilitation from rehabilitation effectiveness: patients receiving HD may retain rehabilitation potential but remain unable to enter a setting in which that potential can be realized.

The poorer acute outcomes observed in the HD group were not fully explained by differences in stroke subtype. Intracerebral hemorrhage accounted for approximately two-thirds of patients receiving HD, and its high mortality in dialysis populations has been consistently reported [10,13,14]. However, maintenance HD remained independently associated with higher in-hospital mortality and lower odds of a favorable Glasgow Outcome Scale outcome after adjustment for age, sex, admission Glasgow Coma Scale score, and stroke subtype. In the propensity score–matched analysis, the association with favorable functional outcome was attenuated and no longer statistically significant, whereas the association with in-hospital mortality remained significant. The direction and magnitude of these associations were maintained after excluding patients with subarachnoid hemorrhage and in the analysis restricted to intracerebral hemorrhage. Previous studies have demonstrated high mortality after intracerebral hemorrhage in patients receiving HD [31], poor outcomes associated with chronic kidney dysfunction [32], and the prognostic importance of cerebral microbleeds [33,34]. These findings suggest that patients receiving maintenance HD constitute a population at high acute risk that cannot be attributed to stroke-subtype distribution alone.

Surgical intervention was performed less frequently in patients receiving HD. However, subarachnoid hemorrhage was less common in this group, and surgical indications for intracerebral hemorrhage depend on hematoma characteristics, neurological severity, and overall medical condition. Because these variables were unavailable, the lower surgical rate should not be interpreted as evidence of less aggressive treatment. Post-acute disposition after stroke is similarly determined by multiple clinical and healthcare-system factors, including neurological recovery, ongoing treatment requirements, family support, and the availability and capabilities of receiving facilities [16,17,18]. Previous studies have described substantial rehabilitation challenges and functional decline among older patients receiving HD [35,36]. Patients receiving maintenance HD frequently require coordination of dialysis schedules and management of hemodynamic instability, fatigue, falls, cognitive impairment, and multiple comorbidities [37,38]. These requirements may substantially limit the number of facilities able to provide intensive rehabilitation while maintaining dialysis.

In Japan, convalescent rehabilitation wards are generally separate from acute-care hospitals, and relatively few are equipped to provide maintenance HD while delivering intensive inpatient rehabilitation [19,20]. The present study identified the inability to secure such a facility as the documented reason for non-rehabilitation transfer in all eligible HD patients who were not transferred. However, this finding does not establish that HD status alone caused the observed difference. Facility acceptance may also have been influenced by medical complexity, staffing, dialysis schedules, bed availability, geographic accessibility, or other factors not fully captured in the medical records. The findings should therefore be interpreted as evidence of a potentially modifiable care-delivery barrier rather than proof of a causal effect of HD itself.

Improving coordination between dialysis and rehabilitation services may represent a practical opportunity to optimize post-stroke care. Multidisciplinary management integrating nephrology, rehabilitation medicine, stroke care, and discharge planning has been associated with improved physical function and greater participation in rehabilitation [39]. In skilled nursing facilities in the United States, more frequent on-site HD has been associated with shorter post-dialysis recovery time [40] and earlier return home [41]. Although healthcare systems differ between countries, these observations suggest that better integration of dialysis and rehabilitation services may expand access to post-acute rehabilitation. Early involvement of nephrology and rehabilitation teams in discharge planning, coordination between dialysis facilities and rehabilitation hospitals, and development of post-acute facilities capable of delivering both services may help reduce this gap. From a healthcare-quality perspective, maintenance HD should therefore be regarded not merely as a comorbidity but as an ongoing treatment requirement that must be incorporated into post-stroke rehabilitation planning to promote continuity and equitable access to care.

### Limitations

This study has several limitations. First, this was a retrospective single-center study conducted at a tertiary emergency care center, which may limit the generalizability of the findings, particularly to patients with milder stroke or those treated in non-tertiary hospitals. Local availability of facilities capable of providing both maintenance HD and inpatient rehabilitation may also differ across regions and healthcare systems.

Second, the number of patients receiving maintenance HD was small, and only two underwent rehabilitation transfer. Consequently, the adjusted estimate for rehabilitation transfer remained imprecise despite the use of Firth’s penalized likelihood logistic regression to reduce small-sample bias associated with sparse outcome data. The additional proxy-defined and sensitivity analyses were exploratory and were not adjusted for multiple comparisons. Their findings should therefore be interpreted cautiously. Furthermore, because the primary analysis was restricted to in-hospital survivors and mortality differed between the groups, survivor bias may have affected group comparability.

Third, rehabilitation eligibility was retrospectively assessed only in patients receiving HD. In the non-HD group, discharge Glasgow Outcome Scale categories after exclusion of patients discharged home were used as proxies for potential rehabilitation eligibility. These proxy definitions may have introduced misclassification and do not permit a direct comparison of eligibility between the groups. In addition, eligibility in the HD group was assessed retrospectively by a single investigator who was aware of the documented clinical course and actual disposition. Inter-rater reliability was not assessed, and hindsight bias cannot be excluded.

Fourth, transfer for continued medical care included both intrahospital and interhospital transfers. Information regarding subsequent rehabilitation, final discharge destination, return home, and long-term functional outcomes was unavailable. Although documented reasons for non-rehabilitation transfer were reviewed in eligible HD patients, the number and characteristics of facilities contacted, individual facility responses, bed availability, geographic accessibility, dialysis schedules, staffing, and details of transfer coordination were not systematically collected. The study therefore could not distinguish the relative contributions of facility availability, facility acceptance, and patient-level medical complexity. Temporal changes in rehabilitation-facility capacity and discharge practices were not systematically recorded; therefore, although rehabilitation transfer rates showed no clear temporal shift across the three study periods, secular changes in the care environment cannot be excluded.

Fifth, pre-stroke modified Rankin Scale scores were available only for patients receiving HD and could not be directly compared with or adjusted for in the non-HD group. Detailed information regarding frailty, sarcopenia, nutritional status, vascular access, dialysis modality and schedule, intradialytic instability, family and social support, and stroke-specific severity measures such as the National Institutes of Health Stroke Scale score and intracerebral hemorrhage volume was also unavailable. Residual confounding by these factors cannot be excluded, and the observed association should not be interpreted as demonstrating that maintenance HD itself caused reduced access to rehabilitation.

Finally, the limited number of patients receiving HD precluded statistically reliable subgroup analyses, including sex-specific analyses.

Despite these limitations, this study consecutively evaluated patients with stroke admitted to a tertiary emergency care center, adjusted acute outcomes for important clinical confounders, and comprehensively assessed post-acute disposition among survivors. Unlike previous studies restricted to patients already admitted to rehabilitation facilities, this study evaluated access to rehabilitation from the acute-care setting and directly examined the discrepancy between retrospective rehabilitation eligibility and actual rehabilitation transfer. Future prospective multicenter studies incorporating patient-, dialysis-, facility-, and healthcare-system-level data are warranted to determine the generalizability of these findings and whether improved coordination between dialysis and rehabilitation services can reduce potentially modifiable barriers to post-acute rehabilitation.

## 5. Conclusions

Maintenance HD was associated with substantially lower transfer to post-acute rehabilitation after stroke, as well as higher in-hospital mortality and poorer functional outcomes. Among 12 patients receiving maintenance HD who were retrospectively considered eligible for post-acute rehabilitation, only 2 were transferred to rehabilitation. For the remaining 10 patients, no receiving facility capable of providing both maintenance HD and inpatient rehabilitation could be secured. These findings suggest a potentially modifiable care-delivery gap in the transition from acute stroke care to rehabilitation. Prospective multicenter studies are needed to determine whether improved coordination and capacity across dialysis and rehabilitation services can expand equitable access to post-acute rehabilitation.

## Figures and Tables

**Table 1:** Baseline characteristics, stroke subtype, and surgical intervention according to maintenance hemodialysis status.

Variable	HD Group	Non-HD Group	*p* Value
Patients, n	54	1119	
Age, years	65.5 [60.0–75.8]	70.0 [56.0–79.5]	0.252
Male sex, n (%)	43 (79.6)	591 (52.8)	<0.001
Systolic blood pressure, mm Hg	190.0 [168.0–220.0]	163.0 [141.0–194.0]	<0.001
Diastolic blood pressure, mm Hg	100.0 [87.0–113.0]	92.0 [78.0–111.0]	0.090
GCS on arrival	6.5 [4.0–12.0]	8.0 [4.0–13.0]	0.313
Stroke subtype, n (%)			<0.001
Cerebral infarction	15 (27.8)	254 (22.7)	
Intracerebral hemorrhage	36 (66.7)	538 (48.1)	
Subarachnoid hemorrhage	3 (5.6)	327 (29.2)	
Surgical intervention, n (%)	20 (37.0)	634 (56.7)	0.005

Data are presented as median [interquartile range] or n (%). Continuous variables were compared using the Mann–Whitney U test. Categorical variables were compared using Pearson’s χ^2^ test or Fisher’s exact test, as appropriate. Systolic blood pressure data were available for 53 of 54 patients in the HD group and 1114 patients in the non-HD group. Diastolic blood pressure data were available for 53 of 54 patients in the HD group and 1105 patients in the non-HD group. The *p* value for stroke subtype represents the overall comparison across the three stroke subtypes. Abbreviations: GCS, Glasgow Coma Scale; HD, hemodialysis.

**Table 2:** In-hospital outcomes and post-acute disposition according to maintenance hemodialysis status.

Variable	HD Group	Non-HD Group	*p* Value
Patients, n	54	1119	
Length of stay, days	11.7 [8.0–21.0]	18.0 [7.0–29.0]	0.101
Favorable GOS outcome, n (%)	15 (27.8)	526 (47.0)	0.007
GOS at discharge, n (%)			<0.001
GR	3 (5.6)	205 (18.3)	
MD	12 (22.2)	321 (28.7)	
SD	16 (29.6)	283 (25.3)	
VS	2 (3.7)	128 (11.4)	
D	21 (38.9)	182 (16.3)	
Discharge destination, n (%)			<0.001
Transfer for rehabilitation	2 (3.7)	299 (26.7)	
Transfer for continued medical care	29 (53.7)	549 (49.1)	
Home discharge	2 (3.7)	89 (8.0)	
In-hospital death	21 (38.9)	182 (16.3)	
In-hospital survivors, n	33	937	
Post-acute disposition, n (%)			<0.001
Transfer to post-acute rehabilitation	2 (6.1)	299 (31.9)	
Transfer for continued medical care	29 (87.9)	549 (58.6)	
Home discharge	2 (6.1)	89 (9.5)	

Data are presented as median [interquartile range (IQR)] or n (%). Length of stay was compared using the Mann–Whitney U test. Favorable GOS outcome was compared using Fisher’s exact test. The overall distributions of GOS at discharge, discharge destination among all patients, and post-acute disposition among in-hospital survivors were compared using exact tests for contingency tables. A favorable GOS outcome was defined as good recovery or moderate disability. Transfer for continued medical care included transfer to another department within the same hospital and transfer to another hospital for purposes other than post-acute rehabilitation. Abbreviations: D, death; GOS, Glasgow Outcome Scale; GR, good recovery; HD, hemodialysis; IQR, interquartile range; MD, moderate disability; SD, severe disability; VS, vegetative state.

**Table 3:** Multivariable logistic regression analyses for in-hospital death and favorable functional outcome.

Variable	In-Hospital Mortality, aOR (95% CI)	*p* Value	Favorable Functional Outcome, aOR (95% CI)	*p* Value
Maintenance HD	4.26 (2.20–8.24)	<0.001	0.38 (0.19–0.77)	0.007
Age, per year	1.02 (1.01–1.03)	0.001	0.97 (0.96–0.98)	<0.001
Male sex	1.02 (0.72–1.46)	0.903	1.01 (0.76–1.35)	0.921
GCS on arrival, per point	0.77 (0.74–0.81)	<0.001	1.29 (1.25–1.33)	<0.001
Cerebral infarction	Reference		Reference	
Intracerebral hemorrhage	1.88 (1.13–3.12)	0.016	0.45 (0.31–0.64)	<0.001
Subarachnoid hemorrhage	2.64 (1.51–4.61)	<0.001	0.60 (0.40–0.90)	0.013

Adjusted odds ratios (aORs) were estimated using multivariable logistic regression models including maintenance HD, age, sex, GCS score on arrival, and stroke subtype. A favorable GOS outcome was defined as good recovery or moderate disability. Abbreviations: aOR, adjusted odds ratio; CI, confidence interval; GCS, Glasgow Coma Scale; GOS, Glasgow Outcome Scale; HD, hemodialysis.

## Data Availability

The datasets generated and/or analyzed during the current study are not publicly available because they contain information that could compromise patient privacy and are subject to institutional restrictions. Data are available from the corresponding author upon reasonable request, subject to approval by the Ethics Committee of Nippon Medical School and applicable institutional requirements.

## Supplementary Materials

The following supporting information can be downloaded at https://www.mdpi.com/article/10.3390/healthcare14193303/s1, Supplementary Table S1: Characteristics and actual disposition of hemodialysis patients considered eligible for post-acute rehabilitation; Supplementary Table S2: Unadjusted effect estimates for post-acute disposition and rehabilitation-transfer analyses; Supplementary Table S3: Rehabilitation transfer according to study period; Supplementary Table S4: Adjusted sensitivity analyses of in-hospital outcomes; Supplementary Table S5: Covariate balance before and after propensity score matching; Supplementary Table S6: Clinical outcomes in the propensity score–matched cohorts.
