# Supplementary material for: Association of Maintenance Hemodialysis with Transfer to Post-Acute Rehabilitation After Stroke: A Single-Center Retrospective Cohort Study
Source: Healthcare (Basel). 2026 Oct 3;14(19):3303. doi: 10.3390/healthcare14193303 (PMC13665153; doi:10.3390/healthcare14193303)
Supplement: Supplementary file 1 [file healthcare-14-03303-s001.zip › healthcare-4541506-supplementary.pdf]

Association of Maintenance Hemodialysis with Transfer to Post-Acute Rehabilitation after Stroke: A Single-Center Retrospective Cohort Study

Supplementary Table S1

Characteristics and actual disposition of hemodialysis patients considered eligible for post-acute rehabilitation

Patient	Age	Sex	Stroke Subtype	Admission GCS	Discharge GOS	Actual disposition
1	80	Female	Cerebral infarction	12	MD	Transfer for continued medical care
2	61	Male	Subarachnoid hemorrhage	14	MD	Transfer for continued medical care
3	63	Male	Cerebral infarction	15	MD	Transfer for continued medical care
4	40	Male	Intracerebral hemorrhage	7	GR	Transfer for continued medical care
5	79	Male	Intracerebral hemorrhage	14	MD	Transfer for continued medical care
6	60	Male	Intracerebral hemorrhage	15	MD	Transfer for continued medical care
7	78	Male	Cerebral infarction	10	MD	Transfer for continued medical care
8	73	Male	Cerebral infarction	13	MD	Transfer for rehabilitation
9	76	Male	Cerebral infarction	13	MD	Transfer for continued medical care
10	73	Male	Cerebral infarction	3	MD	Transfer for continued medical care

11	69	Male	Cerebral infarction	6	MD	Transfer for rehabilitation
12	41	Male	Subarachnoid hemorrhage	12	MD	Transfer for continued medical care

Among the 12 patients receiving maintenance hemodialysis who were considered eligible for post-acute rehabilitation, two patients (16.7%) were transferred for rehabilitation and 10 patients (83.3%) were transferred for continued medical care. For all 10 patients who did not undergo rehabilitation transfer, no facility capable of providing both maintenance hemodialysis and inpatient rehabilitation could be secured.

Abbreviations: GCS, Glasgow Coma Scale; GOS, Glasgow Outcome Scale; GR, good recovery; MD, moderate disability.

Supplementary Table S2

Unadjusted effect estimates for post-acute disposition and rehabilitation-transfer analyses

Outcome	HD group	Non-HD group	Risk ratio (95% CI)	Risk difference, percentage points (95% CI)	p value
Rehabilitation transfer among all patients	2/54 (3.7%)	299/1,119 (26.7%)	0.14 (0.04–0.47)	−23.0 (−26.8 to −13.8)	< 0.001
Rehabilitation transfer among survivors	2/33 (6.1%)	299/937 (31.9%)	0.19 (0.05–0.62)	−25.8 (−31.2 to −12.0)	< 0.001
Continued-care transfer among survivors	29/33 (87.9%)	549/937 (58.6%)	1.50 (1.23–1.66)	29.3 (13.8 to 37.3)	< 0.001
Home discharge among survivors	2/33 (6.1%)	89/937 (9.5%)	0.64 (0.17–2.10)	−3.4 (−8.3 to 10.2)	0.762
GR or MD, excluding home	2/13 (15.4%)	209/441 (47.4%)	0.32 (0.09–0.90)	−32.0 (−44.0 to −4.8)	0.025

discharge					
MD only, excluding home discharge	2/12 (16.7%)	140/310 (45.2%)	0.37 (0.10–1.00)	−28.5 (−41.7 to 0.2)	0.073

Risk ratios compare the HD group with the non-HD group. Confidence intervals for risk ratios were calculated using the score method, and confidence intervals for risk differences were calculated using the Newcombe method.

Abbreviations: CI, confidence interval; GR, good recovery; HD, hemodialysis; MD, moderate disability.

Supplementary Table S3. Rehabilitation transfer according to study period

**Study period**	**HD, n/N (%)**	**Non-HD, n/N (%)**
2015–2017	1/12 (8.3)	135/401 (33.7)
2018–2020	1/10 (10.0)	120/410 (29.3)
2021–2023	0/11 (0.0)	44/126 (34.9)
Overall	2/33 (6.1)	299/937 (31.9)

Data are presented as the number of patients transferred to post-acute rehabilitation divided by the number of in-hospital survivors, with percentages in parentheses.

Abbreviation: HD, hemodialysis.

Supplementary Table S4. Adjusted sensitivity analyses of in-hospital outcomes

Outcome	Analysis population	Patients, n	HD patients, n	HD aOR (95% CI)	p value
In-hospital death	Subarachnoid hemorrhage excluded	843	51	3.98 (1.99–7.97)	< 0.001
Favorable GOS	Subarachnoid hemorrhage excluded	843	51	0.40 (0.19–0.84)	0.016
In-hospital death	Intracerebral hemorrhage only	574	36	4.32 (1.98–9.44)	< 0.001
Favorable GOS	Intracerebral hemorrhage only	574	36	0.27 (0.09–0.82)	0.021

Models included HD status, age, sex, and GCS score on arrival. The analysis excluding subarachnoid hemorrhage was additionally adjusted for stroke subtype.

Abbreviations: aOR, adjusted odds ratio; CI, confidence interval; GCS, Glasgow Coma Scale; GOS, Glasgow Outcome Scale; HD, hemodialysis.

Supplementary Table S5. Covariate balance before and after propensity score matching

Panel A. Overall cohort

**Variable**	**Before matching**			**After matching**		
	HD (n=53)	Non-HD (n=1,114)	SMD	HD (n=53)	Non-HD (n=159)	SMD
Age, years	65.9 ± 11.4	66.9 ± 16.4	0.073	65.9 ± 11.4	66.7 ± 16.1	0.056
Male, n (%)	42 (79.2)	588 (52.8)	0.582	42 (79.2)	126 (79.2)	0.000
GCS	8.2 ± 4.3	8.7 ± 4.4	0.116	8.2 ± 4.3	8.0 ± 4.0	0.058

Variable	Before matching			After matching		
SBP, mmHg	192.9 ± 46.1	168.6 ± 40.7	0.560	192.9 ± 46.1	192.7 ± 43.9	0.004
Cerebral infarction, n (%)	14 (26.4)	252 (22.6)	0.088	14 (26.4)	42 (26.4)	0.000
ICH, n (%)	36 (67.9)	536 (48.1)	0.410	36 (67.9)	108 (67.9)	0.000
SAH, n (%)	3 (5.7)	326 (29.3)	0.654	3 (5.7)	9 (5.7)	0.000

Panel B. In-hospital survivors

Variable	Before matching			After matching		
	HD (n=32)	Non-HD (n=934)	SMD	HD (n=32)	Non-HD (n=96)	SMD
Age, years	66.1 ± 12.2	66.4 ± 16.3	0.020	66.1 ± 12.2	65.9 ± 15.0	0.011
Male, n (%)	26 (81.2)	496 (53.1)	0.628	26 (81.2)	81 (84.4)	0.083
GCS	9.8 ± 4.1	9.3 ± 4.3	0.123	9.8 ± 4.1	9.6 ± 4.6	0.050
SBP, mmHg	191.2 ± 44.1	167.6 ± 38.8	0.569	191.2 ± 44.1	189.2 ± 42.1	0.044

**Variable**	**Before matching**			**After matching**		
Cerebral infarction, n (%)	12 (37.5)	231 (24.7)	0.278	12 (37.5)	36 (37.5)	0.000
ICH, n (%)	19 (59.4)	446 (47.8)	0.235	19 (59.4)	57 (59.4)	0.000
SAH, n (%)	1 (3.1)	257 (27.5)	0.720	1 (3.1)	3 (3.1)	0.000

Data are presented as mean ± standard deviation or n (%). Propensity scores were estimated using age, sex, GCS, stroke subtype, and SBP. Patients were matched using 1:3 nearest-neighbor matching without replacement, with a caliper width of 0.2 standard deviations of the logit of the propensity score and exact matching on stroke subtype. An absolute SMD <0.10 was considered indicative of adequate covariate balance.

Abbreviations: GCS, Glasgow Coma Scale; HD, hemodialysis; ICH, intracerebral hemorrhage; SAH, subarachnoid hemorrhage; SBP, systolic blood pressure; SMD, standardized mean difference.

Supplementary Table S6. Clinical outcomes in the propensity score–matched cohorts

Population	Outcome	HD	Non-HD	Matched OR (95% CI)	p value
Overall matched cohort	In-hospital mortality	21/53 (39.6%)	31/159 (19.5%)	2.71 (1.36–5.42)	0.005
Overall matched cohort	Favorable GOS outcome	14/53 (26.4%)	60/159 (37.7%)	0.53 (0.24–1.13)	0.101
Matched in-hospital survivors	Rehabilitation transfer	2/32 (6.3%)	25/96 (26.0%)	0.20 (0.04–0.87)	0.032

Data are presented as n (%). Matched ORs were estimated using conditional logistic regression accounting for matched sets. A favorable GOS outcome was defined as good recovery or moderate disability. The overall matched cohort was used for analyses of in-hospital mortality and favorable GOS, whereas the rehabilitation-transfer analysis was restricted to in-hospital survivors.

Abbreviations: CI, confidence interval; GOS, Glasgow Outcome Scale; HD, hemodialysis; OR, odds ratio.
